# Molecular Recognition of the HPLC Whelk-O1 Selector towards the Conformational Enantiomers of Nevirapine and Oxcarbazepine

**DOI:** 10.3390/ijms22010144

**Published:** 2020-12-25

**Authors:** Roberta Franzini, Marco Pierini, Andrea Mazzanti, Antonia Iazzetti, Alessia Ciogli, Claudio Villani

**Affiliations:** 1 Department of Chemistry and Technology of Drugs, “Department of Excellence 2018−2022”, Sapienza University of Rome, Piazzale Aldo Moro 5, 00185 Rome, Italy; marco.pierini@uniroma1.it (M.P.); antonia.iazzetti@uniroma1.it (A.I.); alessia.ciogli@uniroma1.it (A.C.); 2 Dipartimento di Chimica Industriale “Toso Montanari”, Università di Bologna, V. Risorgimento 4, 40136 Bologna, Italy; andrea.mazzanti@unibo.it

**Keywords:** chiral molecular recognition, chirality, enantioselective HPLC, molecular docking, VT-NMR, conformational chirality, stereodynamics, dynamic chromatography, tricyclic drugs

## Abstract

The presence of stereogenic elements is a common feature in pharmaceutical compounds, and affording optically pure stereoisomers is a frequent issue in drug design. In this context, the study of the chiral molecular recognition mechanism fundamentally supports the understanding and optimization of chromatographic separations with chiral stationary phases. We investigated, with molecular docking, the interactions between the chiral HPLC selector Whelk-O1 and the stereoisomers of two bioactive compounds, the antiviral Nevirapine and the anticonvulsant Oxcarbazepine, both characterized by two stereolabile conformational enantiomers. The presence of fast-exchange enantiomers and the rate of the interconversion process were studied using low temperature enantioselective HPLC and VT-NMR with Whelk-O1 applied as chiral solvating agent. The values of the energetic barriers of interconversion indicate, for the single enantiomers of both compounds, half-lives sufficiently long enough to allow their separation only at critically sub-ambient temperatures. The chiral selector Whelk-O1 performed as a strongly selective discriminating agent both when applied as a chiral stationary phase (CSP) in HPLC and as CSA in NMR spectroscopy.

## 1. Introduction

The chemistry of chirality is a perfect example of multidisciplinary science, and it has inspired interest for many decades, recently taking advantage of modern analytical and computational techniques. Chirality is a common property in bioactive compounds and a high percentage of the drugs available on the market, including some of the most prescribed ones, and is formulated using only a single enantiomer, thus requiring additional challenges during the developmental process [1,2,3]. While configurational chirality, which derives from the presence of a classical stereogenic center, is easily detected during the drug design process, conformational chirality is sometimes overlooked despite its frequent recurrency [4]. Conformational enantiomers interconvert only by rotation around single bonds or a ring reversal mechanism, with a rate that is temperature- and time-dependent. Furthermore, the rotation can be somehow slow enough to consider them stereochemically stable at ambient temperature, in which case they are defined as atropoisomers [5,6]. Conformational enantiomers can interconvert on very different timescales, and this must be considered carefully during a design process in order to obtain safe and effective drugs [7]. For both configurational and conformational enantiomers, pharmacological and pharmacokinetic properties may be very different; thus, the therapeutical index or the adverse effects of two enantiomeric species can vary dramatically [8,9,10,11,12]. Consequently, the demand for methodologies to thoroughly characterize atropoisomers and provide conformationally pure chiral compounds has increased in recent decades, pushing towards a progressive boosting of separation sciences and in particular of chromatographic techniques such as HPLC with chiral stationary phases (CSP-HPLC). In recent years, the development of novel chiral stationary phases (CSPs) for high performance liquid chromatography and technological innovations have made HPLC a potential choice for the separation of conformational enantiomers [13,14]. Moreover, in particular when coupled to other techniques such as NMR or circular dichroism spectroscopy, CSP-HPLC is a valid support in the comprehension of the stereomutation process of atropoisomers and in the assignment of the absolute configuration [15,16,17,18,19,20,21]. At present, there is a vast choice of CSPs that can be applied for the separation of enantiomers on both the analytical and preparative scale, and they can be constituted by a well-defined chiral molecule or by a natural or synthetic polysaccharide [22,23,24]. Molecular modeling and related computational techniques represent an essential tool to elucidate the mechanisms and restrictions of the chromatographic separation process, and results are perfectly suitable for those CSPs constituted by small organic molecules such as the brush-type CSP [25,26,27]. The study of chiral molecular recognition mechanisms [28] is particularly interesting for the understanding and improvement of chromatographic enantioseparations, and the selector–selectand relationship can be conveniently studied by direct chromatographic methods [29] or by docking methodologies with a step procedure that takes into account some fundamental parameters such as the conformational flexibility of both species, the electrostatic and Van Der Waals interactions and the solvation effects [30]. The docked diastereomeric complexes generated by the enantioselective interaction of a chiral analyte with a selector are considered when calculating the thermodynamic data, in particular the ΔΔG, which can be used to predict elution order and selectivity factor. Small molecule chiral selectors, in their soluble forms, can also be conveniently applied as chiral solvating agents in NMR spectroscopy, and the formation of the host–guest systems in solution can be used to measure enantiopurity, to assign absolute configuration or to study stereomutation processes by means of variable temperature NMR (VT-NMR) experiments [31,32,33].

**Figure 1:**
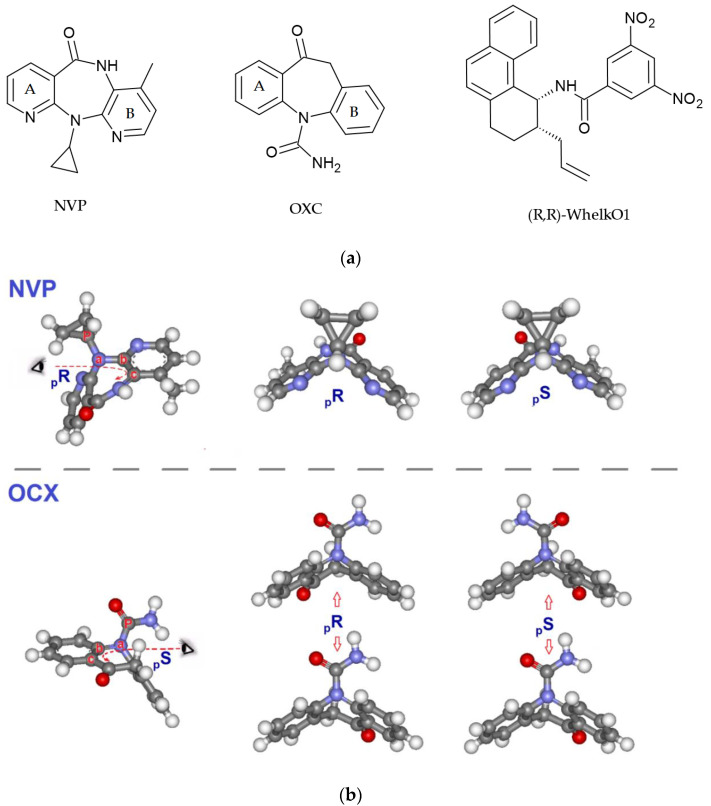
(**a**) Chemical structures of Nevirapine (NVP), Oxcarbazepine (OXC) and the soluble analogue of the chiral selector (*R*,*R*) Whelk-O1. (**b**) (*pR*) and *(pS*) conformational enantiomers of NVP and OXC. (**b**) With reference to the molecules of **NVP** and **OXC**, in the figure, the atom outside of but closest to the chiral plane (the “pilot atom”) has been assigned the letter ***P***. Starting from ***P***, the three atoms closest to it and lying on the plane, chosen according to CIP rules, have been assigned the letters ***a***, ***b*** and ***c***. When the succession of the ***a***, ***b*** and ***c*** atoms in the chiral plane forms a clockwise array, the assigned configuration is ***pR***, while if the array is counterclockwise, the assigned configuration is ***pS***.

Herein, we describe a study, by docking analysis, of the recognition mechanism between the chiral HPLC selector Whelk-O1 and the fast-exchange enantiomeric conformations of two different pharmaceutical compounds, the anti-HIV drug Nevirapine and the antiepileptic Oxcarbazepine (Figure 1). Compounds featuring a non-planar seven-membered ring in their molecular backbone are known to be chiral, since the absence of planarity allows the existence of two, sometimes fast-exchange conformational enantiomers that interconvert due to a ring-flip mechanism, typical of dibenzoazepinic and diazepinic systems [34,35,36]. The chiral HPLC selector Whelk-O1 was developed in the 1990s by Pirkle and coworkers following the reciprocity concept [37] and has become popular for its selectivity towards configurational and conformational enantiomers of a wide range of compounds. The molecular recognition mechanism of Whelk-O1 has been the object of many studies that clarified the importance of hydrogen bonding and π–π stacking in the interaction with an analyte [38,39]. The ability of the chiral selector to discriminate the enantiomers of the studied compounds is exploited both by variable temperature HPLC (VT-HPLC) and variable temperature NMR (VT-NMR).

## 2. Results and Discussion

### 2.1. Chromatographic Retention and Enantioselectivity

A chiral stationary phase derivatized with the synthetic selector (*R*,*R*)-whelk-O1 was chosen to start a screening in order to find the best fitting chromatographic condition to demonstrate the existence of two conformational enantiomers for both NVP and OXC and to measure the relative enantiomerization barriers by VT-HPLC.

**Figure 2:**
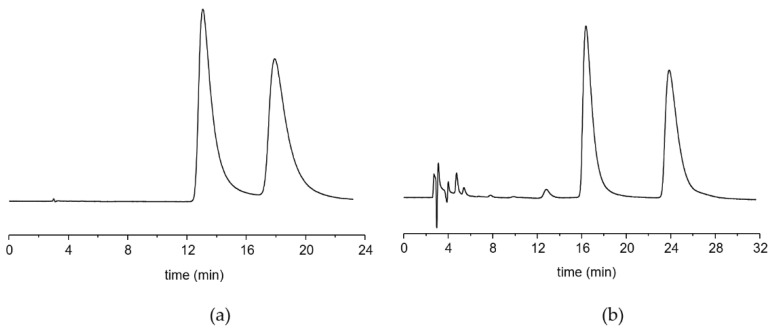
Experimental chromatograms showing two peaks corresponding to the enantiomeric conformations of (**a**) Nevirapine and (**b**) Oxcarbazepine obtained at −50 °C on a (*R*,*R*)WhelkO1 CSP.

Using a standard analytical column maintained at a controlled temperature of 25 °C, both compounds were eluted as a single broad peak. Therefore, to prove that the lack of resolution was imputable to a fast interconversion process, the temperature of the column was decreased to −50 °C. In these conditions, two peaks with a 50:50 (% area) ratio, corresponding to the two conformational enantiomers of NVP, are well resolved at the baseline, despite a significant tailing (Figure 2a). Analogously, two baseline-resolved peaks are evident for OXC at the same temperature (Figure 2b). Compounds featuring a non-planar seven-membered ring in their molecular backbone are known to be chiral, since the absence of planarity allows the existence of two, sometimes fast-exchange conformational enantiomers.

Retention factors and selectivity for both compounds in the experimental conditions are reported in Table 1. As expected, the WhelkO1 CSP was sufficiently selective towards the enantiomers of NVP and OXC with an eluent composed of hexane and methyl chloride in a 50:50 (*v*/*v*) proportion and the addition of 2% methanol for NVP and 5% methanol for OXC.

### 2.2. Variable Temperature HPLC and Enantiomerization Barriers

Dynamic high performance liquid chromatography on enantioselective stationary phases is a well-established technique to investigate chiral molecules with labile stereogenic elements that result in stereoinversion processes occurring on the timescale of the HPLC separation [40,41,42]. Kinetic parameters for on-column interconversion can be extracted from exchange-deformed experimental peak profiles with computer simulation.

**Figure 3:**
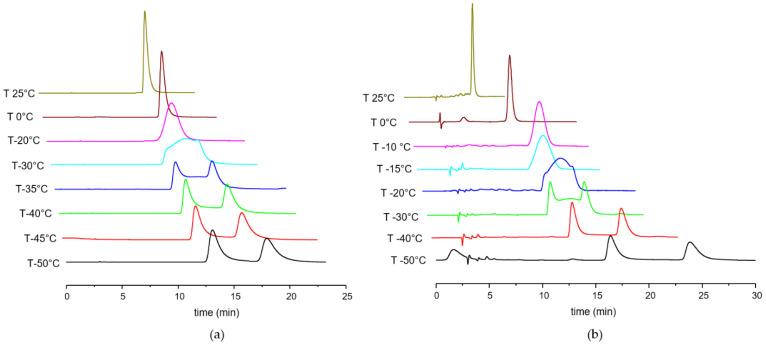
VT-HPLC elution profile registered for (**a**) NVP and (**b**) OXC. All the temperatures must be considered with a ±1 °C deviation.

The technique has been used at a wide range of temperatures and is complementary in scope to dynamic nuclear magnetic resonance spectroscopy. The potential of the method has been demonstrated in recent years, with applications in the analysis of chiral compounds featuring either fast or slow internal motions leading to chirality inversion. For both compounds the energetic barriers of enantiomerization have been derived from the analysis of the fast-exchange chromatographic profiles observed performing low temperature HPLC [43] on the chiral stationary phase (*R*,*R*)-WhelkO1. By increasing the temperature of the chromatographic column in a range between −50 and 25 °C, a progressive coalescence of the peaks into a single broad peak could be observed for both compounds. Indeed, this is representative of an on-column enantiomerization that takes place on a timescale comparable to the one of the elution process and thus can be conveniently studied by VT-HPLC. The results show only very small differences between the elution profiles of the two compounds, which present a similar temperature-dependent trend (Figure 3).

At ambient temperature, a single peak is observed for NVP, indicating a fast interconversion between the two enantiomeric conformers, whereas at lower temperatures, progressive broadening and eventual characteristic plateau-shaped elution profiles are evident. Lastly, at −50 °C the isomerization process slowed down enough to reveal two well-resolved peaks.

At intermediate temperature, the presence of characteristic plateau-shaped profiles indicates a dynamic on-column interconversion process. Dynamic elution profiles of NVP registered at −35 and −45 °C were simulated with the software Auto-D-HPLC-Y2K [41,42,44,45,46,47,48,49,50,51,52,53,54,55,56,57,58,59,60,61,62,63,64,65,66,67,68,69] to obtain the enantiomerization barriers. 

The computational software is based on the stochastic model and starts from experimental parameters, such as the retention factors and plate numbers, to calculate the apparent rate constants ka1,2 and ka2,1 for the interconversion process. These constants are the result of the weighted average between the rate constant of the isomerization process in the mobile phase (km) and in the stationary phase (ks), which takes place in both directions, respectively, from the first eluted (1,2) towards the second eluted and the other way round (2,1). The rate constants in the mobile and stationary phase slightly differ from each other due to a retarding effect induced by the CSP, particularly evident on the second eluted conformational enantiomer, which spends more time in the binding with the stationary phase.

Calculations were repeated iteratively until the software generated the best fitting simulated chromatogram. Eventually, introducing the value of the apparent rate constants into the Eyring equation and considering a transmission factor of 1, a ΔG^ⱡ^_1,2_ of 17.01 kcal/mol and a ΔG^ⱡ^_2,1_ of 17.14 kcal/mol at −35 °C were calculated for NVP. The energetic barrier measured for the enantiomerization of the conformational enantiomers of NVP almost matches the one reported in the literature and calculated by computational methods and variable temperature NMR analysis.

VT-HPLC elution profiles of OXC show a similar trend, and distorted plateau-shaped elution profiles are clearly seen at −20 and −30 °C, while at −40 °C the interconversion is still detectable, but the de-coalescence is almost complete. The apparent rate constants at the experimental temperatures were measured from the simulated dynamic chromatograms of OXC and were used to calculate a ΔG^ⱡ^_1,2_ of 17.55 kcal/mol and a ΔG^ⱡ^_2,1_ of 17.68 kcal/mol at −20 °C.

Table 2 shows the values of the activation energies calculated at different experimental temperatures for the enantiomerization process of both compounds (see Supplementary Materials for simulated and experimental chromatograms).

As expected, considering the structural analogy between the two compounds and the common feature responsible for the potential conformational chirality, the energetic barrier of enantiomerization of NVP and OXC is quite similar. 

Further calculations were also carried out in order to confirm the experimental energetic barrier, which, at room temperature, is opposed to the interconversion between the couple of enantiomers (*pR*)-NVP/(*pS*)-NVP, as well as between the couple (*pR*)-OXC/(*pS*)-OXC. For each of the NVP and OXC species, such evaluations were performed in four steps:

- Semiempirical AM1 level of calculation of the energetic profile that leads to the change of configuration of such compounds, from the initial (*pR*) to the final (*pS*) condition;
- Optimization performed at the ab initio B3LYP/6−31G* density functional level of theory (DFT), of the transition state (TS) geometries of the enantiomerization processes suggested by the energy profile achieved in the previous step;
- The ground states (GS) of NVP and OXC obtained by the conformational search described in Section 2.4.1: “Conformational Analysis of NVP and OXC”;
- Single point energy calculations performed at the wB97X-D/6-311++G** level of theory on the GS and TS geometries obtained for NVP and OXC in the second step.

The obtained activation energies ΔE^#^ for the enantiomerization processes of NVP and OXC were 15.9 and 16.9 kcal mol^−1^, respectively. Interestingly, such results appear entirely consistent with the relevant experimental data obtained, by D-HPLC determinations, at very low ranges of temperature (between −30 and −40 °C). Indeed, the ΔG^#^ values extrapolated at the temperature of 25 °C starting from the experimental ones (three for both NVP and OXC, see Table 2) were 15.6 and 17.3 kcal mol^−1^, respectively. From the geometrical point of view, as the single enantiomers move along the reaction coordinate for the enantiomerization process, the angle between the planes containing the aromatic rings widens, and the methyl group of NVP and the carbonyl oxygen of OXC come closer to the peri hydrogen of the nearby aromatic ring, eventually leading to severe steric repulsion in the transition state structures.

### 2.3. Variable Temperature NMR

Variable temperature nuclear magnetic resonance (VT-NMR) has been used to measure the enantiomerization barriers of both NVP and OXC and give a solid support to the data obtained by VT-HPLC. The possibility to confirm an isomerization energetic barrier with more than one technique should be always taken into consideration, since it gives more solidity to the data. NMR can be considered as a complementary technique, ensuring reliable HPLC results. In particular, VT-NMR is well suited for the study of the stereomutation processes of stereolabile compounds and is applicable within an even larger range of activation energies of enantiomerization when compared to VT-HPLC [70].

#### 2.3.1. VT-NMR Nevirapine in the Presence of (R,R)-Whelk-O1 as Chiral Solvating Agent

The progressive coalescence of the signals relative to the diastereomeric methylene protons of the cyclopropyl portion of NVP has been studied by Clayden and coworkers [71] to measure the energetic barrier of enantiomerization. However, the spin system associated with the cyclopropyl under fast exchange conditions determines the presence of multiplets that can lead to misleading interpretations. Thus, considering the good selectivity of the chromatographic selector (*R*,*R*)-WhelkO1, we employed it as a chiral solvating agent in NMR.

**Figure 4:**
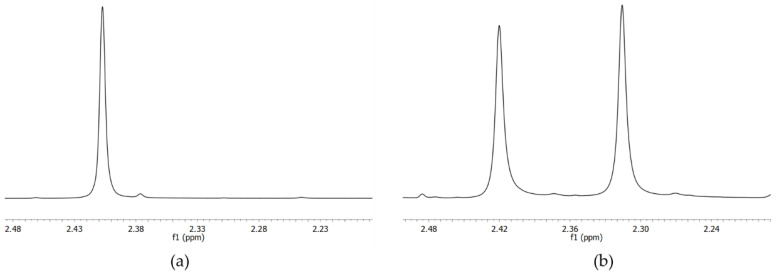
(**a**) ^1^HNMR signal corresponding to the methyl protons in Nevirapine and (**b**) in the mixture 1:1 with the CSA Whelk-O1 (400 MHz in CDCl_3_).

The soluble analogue of the HPLC selector (*R*,*R*)-Whelk-O1 (see Figure 1) was added in a stochiometric amount to a solution of Nevirapine 4 × 10^−3^ M in CDCl_3_, and the formation of two diastereoisomeric abducts resulting from the reversible association between the analyte and the chiral solvating agent was detected by the splitting of the singlet corresponding to the methyl group (Figure 4).

The two signals have a difference of chemical shift (Δδ) of 0.1 ppm corresponding to a 40 Hz separation and show partial de-racemization as indicated by an integral ratio of 1:1.13. Saturating the solution with the chiral solvating agent, the separation increases up to 60 Hz, along with the integral ratio, which changes to 1:1.18.

**Figure 5:**
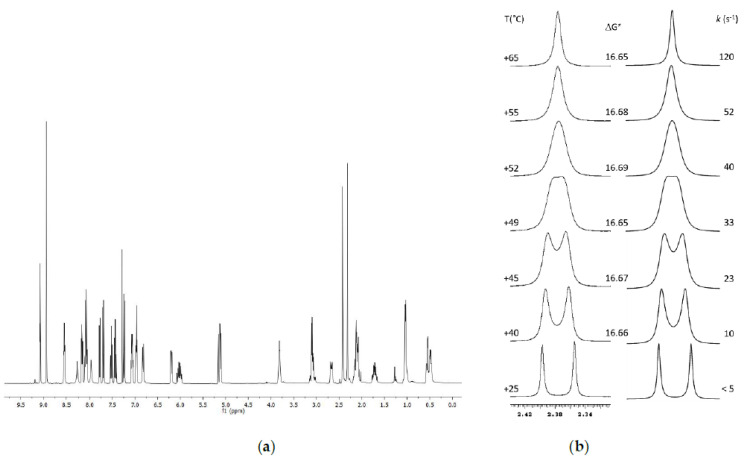
(**a**) ^1^H-NMR of an equimolar solution of (R,R)-WhelkO1 and Nevirapine in CDCl_3_; (**b**) progressive coalescence of the methyl group signals monitored by VT-NMR.

Upon raising the temperature, the two diastereomeric adducts start to exchange, and the coalescence point is reached at +49 °C. (Figure 5), eventually yielding a single signal above +52 °C. The line shape analysis was performed with a PC program implementing the QCPE code [72]. The rate constant at each temperature was determined by matching the simulated with the experimental spectrum, and the free energy of activation (ΔG^ⱡ^) could then be derived at each temperature by means of the Eyring equation. The racemization barrier obtained for Nevirapine was 16.7 kcal/mol, a value consistent both with that derived from dynamic chromatography experiments and that reported in the literature and measured by VT-NMR.

#### 2.3.2. VT-NMR of Oxcarbazepine

The enantiomerization barrier of OXC was also confirmed by VT-NMR, studying the fast exchange profile of the diastereotopic protons of the methylene in the seven-membered ring. Measurements were performed in a range between +30 and +133 °C. At the coalescence temperature (+112 °C), the kinetic rate of interconversion was identified by line shape analysis, providing a ΔG^ǂ^ = 17.7 kcal/mol (see Supplementary Materials for VT-NMR spectra). Notably, in our system we obtained overlapping results by DNMR and by DHPLC, in spite of the different temperature ranges explored and the different solvents used, suggesting a negligible entropy of activation for the stereomutation process. Additionally, no perturbing effects were observed on the enantiomerization barriers by the chiral solvating agent (DNMR of NVP) or by the chiral stationary phase (DHPLC of NVP and OXC). The experimental values of the enantiomerization barriers are also in good agreement with the DFT calculated data. The whole set of data is shown in Table 3, where DG^‡^_1→2_ and ΔG^‡^_2→1_ are the HPLC derived barriers for the conversions of the first into the second eluted enantiomer, and vice versa. As can be seen, the absolute differences between the two experimental methods (ΔΔG^‡^ HPLC/NMR ~0.1–0.2 kcal/mol for NVP and 0.02–0.15 kcal/mol for OXC) are within the experimental error range.

### 2.4. Computational Analysis of the Host–Guest Realtionship between Whelk-O1 and NVP/OXC

#### 2.4.1. Conformational Analysis of NVP and OXC

Semiempirical calculations in vacuo, based on the functional AM1, were employed to conduct conformational searches on the *pR* enantio-pure structures of both NVP and OXC. Next, the obtained conformations were all further optimized at the HF/3-21G level of theory, which, within a 3 kcal mol^−^^1^ energy window, afforded just one geometry for NVP and two conformations for OXC (OXC1 and OXC2), though this was different for the dihedral angle involving the connection of the H_2_N-C=O fragment to the nitrogen atom of the tricyclic structure (i.e., the O=C group of the quoted fragment in sin or anti disposition with respect to the other C=O group bonded to the epta-azacycle). Both guest molecules show a bent conformation, with the planes containing the outer aromatic rings forming an angle of 58.13° for NVP and 57.13° for OXC.

#### 2.4.2. “Quasi Flexible” Docking Analysis of the Host–Guest System

Such *pR* geometries, as well as their corresponding *pS* enantiomers, were then employed as guest molecules to perform “quasi-flexible” automatic docking [44] against the X-ray structure of the (*S*,*S*) chiral selector (W-O1), used as the common host, which constitutes the stationary phase of the well-known Welk-O1 columns (the host–guest approach options set were: 52 directions of translation and 272 relative orientations of guest to host for each couple of host–guest conformations, giving so rise to the generation of 14,040 geometries of complexes).

The salient features of the structure are the presence of a cleft formed by the naphthyl and dinitrobenzoyl rings (at an angle of 75.78°) and the presence of the carboxamide NH group within the cleft as potential site for H-bond interaction. 

**Figure 6:**
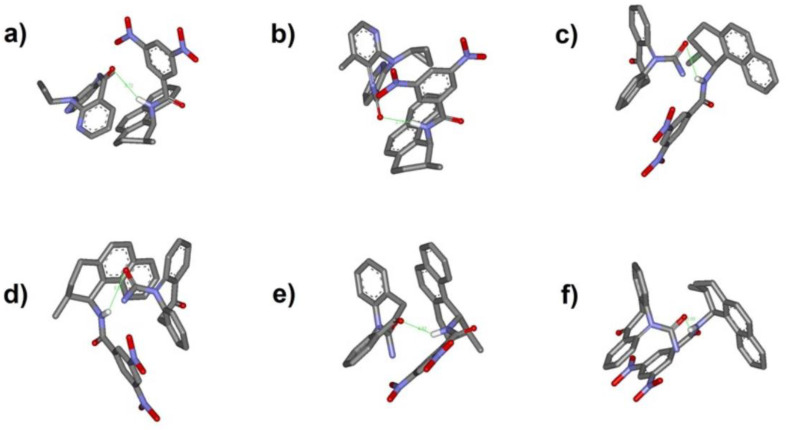
Structures of the host–guest complexes investigated with molecular docking. Hydrogen, except for the NH of the host, is omitted for clarity. (**a**) (pR)-NVP:host; (**b**) (pS)-NVP:host; (**c**) (pR)-OXC1:host; (**d**) (pS)-OXC1:host; (**e**) (pR)-OXC2:host; (**f**) (pS)-OXC2:host.

The achieved diastereomeric ensembles of adducts, generated using the molecular mechanics force field MM2 implemented within the computer program MolInE [30], after suitable selection based on both energetic and geometric criteria, were next optimized by fully relaxing their structure with the Batchmin program, again resorting to the molecular mechanics MM2* Force Field. For the (*pR*)-NVP:(*S*,*S*)W-O1 and (*pS*)-NVP:(*S*,*S*)W-O1 adducts, the docking simulation indicated that the homochiral (*pS*)-(*S*,*S*) complex is more stable than its diastereomeric counterpart (*pR*)-(*S*,*S*) by 0.64 kcal mol^−1^. Regarding the diastereomeric (*pR*)-OXC:(*S*,*S*)W-O1 and (*pS*)-OXC:(*S*,*S*)W-O1 adducts, it was found that the hetero-chiral (*pR*)-(*S*,*S*) complex achieves a slightly greater stability, with a difference between the calculated host–guest interaction energies amounting to just 0.22 kcal mol^−1^. These numbers compare well with the HPLC derived enantioselectivity of ΔΔG = 0.17 kcal/mol and ΔΔG = 0.19 kcal/mol for NVP and OXC, respectively, obtained from the relationship ΔΔG = −RTln α, where ΔΔG is the difference in the free energy of interaction between the two enantiomers and the CSP, R is the gas constant, T is the absolute temperature of the column and α is the chromatographic enantioselectivity (see Table 1). Inspection of the docked structures (Figure 6) reveals that the (*pR*)-NVP guest molecule places its ring over the host naphthyl ring and directs the B ring towards the host DNB ring in a face-to-edge fashion. On the other hand, (*pS*)-NVP is found within the cleft of the host, with the A and B rings aligned over the host naphthyl and DNB rings, respectively. In the complex with (*pR*)-OXC1, the guest adopts an outer complexation geometry, with its B ring stacked parallel to the host DNB ring and the A ring facing the ethylene bridge of the host. (*pS*)-OXC1, (*pR*)-OXC2 and (*pS*)-OXC2 are all placed inside the host cleft, with their B ring stacked on the DNB host ring and the A ring aligned over the host naphthyl ring. In all the host–guest complexes, the carbonyl oxygen on the ring of NVP and OXC is at a hydrogen bonding distance with the amide NH of the host. The distances found in the complexes of the host with the guests are for (*pR*)-NVP 3.13 Å, (*pS*)-NVP 2.12 Å, (*pR*)-OXC1 3.25 Å, (*pS*)-OXC1 3.28 Å, (*pR*)-OXC2 3.09 Å and (*pS*)-OXC2 4.84 Å. It is interesting to note that the shorter H-bond distances are always found in the more stable host–guest complexes.

## 3. Materials and Methods

### 3.1. Chemicals and Materials

All solvents were HPLC grade purity and were purchased from Sigma-Aldrich (St. Louis, MO, USA). Nevirapine was purchased from Sigma-Aldrich (St. Louis, MO, USA). Oxcarbazepine was obtained from a tablet containing 300 mg of active compound. The tablet was triturated, the active compound was extracted with CH_3_CN and after evaporation of the solvent the chemical purity was checked by 1H-NMR and HPLC, resulting in a measurement of more than 90%. The HPLC selector (*R*,*R*)-Whelk-O1 was a gift from Regis Technologies Inc. (Morton Grove, IL, USA) Deuterated solvents for NMR were purchased from Sigma-Aldrich (St. Louis, MO, USA).

### 3.2. Computational Analysis

All calculations performed at semiempirical (functional AM1), Hartree–Fock (3-21G basis set) and DFT level of theory (geometry optimizations, modeling of transition states and single point evaluations) were carried out by means of the computer program Spartan 10v110. The DFT optimization of the GS and TS geometries of NVR and OXA was carried out at the B3LYP/6-31G* level of theory. At the same level of theory, frequency calculations were also performed for the optimized TS structures, in order to ascertain the existence of only one imaginary frequency (i48 for NVR and i41 for OXA). Single point evaluations were obtained at the ωB97X-D/6-311++G** level of theory. Rigid dockings between the X-ray structure of the (*S*,*S*)-Whelk-O1 host and the NVP and OXC guest molecules were performed using the homemade computer program MolInE. The host–guest approach options set were: 52 directions of translation and 272 relative orientations of guest to host for each couple of host–guest conformations. Molecular mechanics optimizations of all the adduct geometries obtained by a rigid docking algorithm were performed by using the Batchmin computer program, with the following options set: MM2* Force Field, PR conjugate gradient minimization.

### 3.3. HPLC Measurements

#### 3.3.1. Chromatographic Apparatus and Column

Analytical chromatography was performed using a Jasco (Tokyo, Japan) HPLC system with a universal Rheodyne 20 μL injector and Jasco PU 980 pump. Detection was conducted using a Jasco UV975 detector. Low temperature dynamic HPLC experiments were performed using a homemade cooling device and a cooling mixture composed of acetone and dry ice.

The CSP (*R*,*R*)-Whelk-O1 (250 × 4.6 mm L × I.D., 5 µm particle size) used to perform VT-HPLC experiments was purchased from Regis Technologies Inc. Samples for the analysis were prepared by dissolving 1 mg in 1 mL of mobile phase.

#### 3.3.2. Simulation of Dynamic Chromatograms

Simulations of variable temperature experimental chromatograms presenting a dynamic profile were performed with Auto DHPLC y2k (Auto Dynamic HPLC) (Rome, Italy), using the stochastic model. Both chromatographic and kinetic parameters can be automatically optimized by a simplex algorithm until the best agreement between experimental and simulated dynamic chromatograms is obtained.

### 3.4. NMR Measurements

1H-NMR spectra were recorded at 400 MHz on a Bruker 400 NMR spectrometer and at 600 MHz (VT-NMR) on a Varian Unity-INOVA 600. The solutions used for the NMR spectra had a 0.04 M concentration. Variable temperature 1H-NMR spectra were acquired without spinning using a 5 mm dual direct probe with a 9000 Hz sweep width, 3.0 µs pulse width, 3 s acquisition time and 1 s delay time. Temperature calibrations were performed using a digital thermometer and a Cu/Ni thermocouple placed in an NMR tube filled with 1,1,2,2-tetrachloroethane. The uncertainty in temperature measurements of the samples can be estimated as ±1 °C.

## Figures and Tables

**Table 1:** Retention factors (k) and selectivity (α).

Compound	T Column °C	k’_1,2_	k’_2,1_	α
NVP ^1^	−50	3.36	4.99	1.48
OXC ^2^	−50	4.47	6.95	1.55

^1^ mobile phase: hexane/dichloromethane/methanol 50:50:2 (*v*/*v*); ^2^ mobile phase: hexane/dichloromethane/methanol 50:50:5 (*v*/*v*).

**Table 2:** Activation energies of enantiomerization at different temperatures.

Compound	T Column °C	ΔG^ⱡ^_12_ kcal/mol	ΔG^ⱡ^_21_ kcal/mol
NVP	−30	16.82	16.96
	−35	17.01	17.14
	−40	17.08	17.22
OXC	−20	17.55	17.68
	−30	17.30	17.42
	−40	17.39	17.53

Temperatures should be considered ±0.1 °C and ΔG^ⱡ^ ± 0.02 kcal/mol.

**Table 3:** Comparison of activation free energies (ΔG^‡^) for the interconversion process obtained by different techniques.

Compound	ΔG^‡^ NMR (kcal/mol)	T (°C)	ΔG^‡^ HPLC (kcal/mol)	T (°C)	ΔG^‡^ DFT (kcal/mol)	T (°C)
Nevirapine (NVP)	16.7	49–52	ΔG^‡^_1→2_ 16.82	−30	15.9	25
			ΔG^‡^_2→1_ 16.96	−30		
Oxcarbazepine (OXC)	17.7	33–133	ΔG^‡^_1→2_ 17.55	−30	16.9	25
			DG^‡^_2→1_ 17.68	−30		

## Supplementary Materials

Supplementary materials can be found at https://www.mdpi.com/1422-0067/22/1/144/s1.
