# Supplementary material for: Molecular Recognition of the HPLC Whelk-O1 Selector towards the Conformational Enantiomers of Nevirapine and Oxcarbazepine
Source: Int J Mol Sci. 2020 Dec 25;22(1):144. doi: 10.3390/ijms22010144 (PMC7796420; doi:10.3390/ijms22010144)
Supplement: Supplementary file 1 [file ijms-22-00144-s001.pdf]

**Supporting information**

**1.NMR spectra**

$^1$HNMR nevirapine

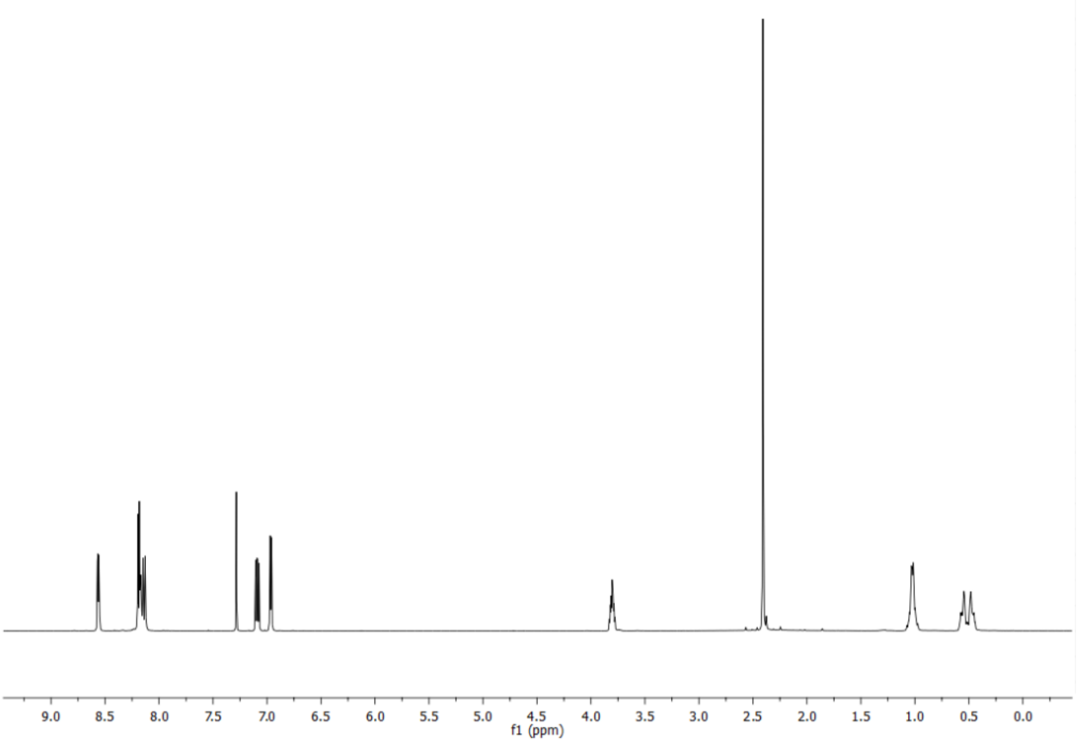


$^1$HNMR (R,R)WhelkO1

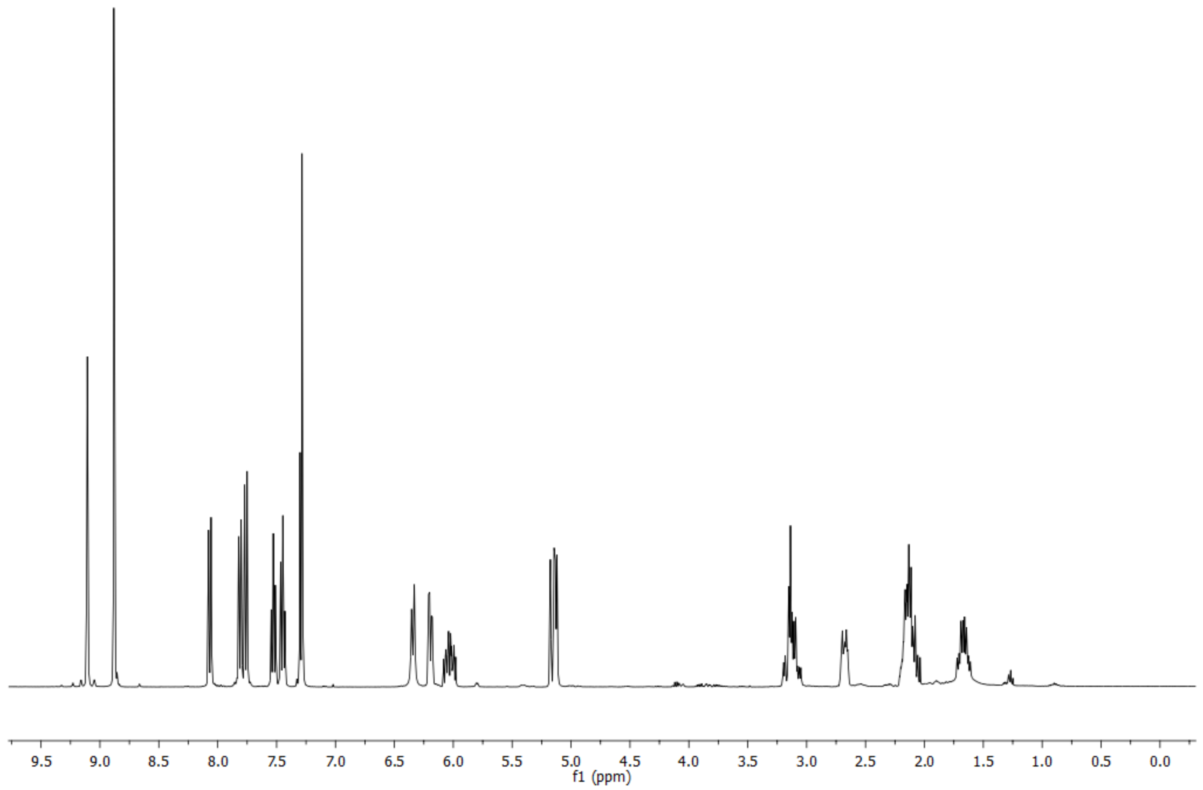


$^{1}$HNMR Nevirapine + saturated solution of (R,R)whelk01

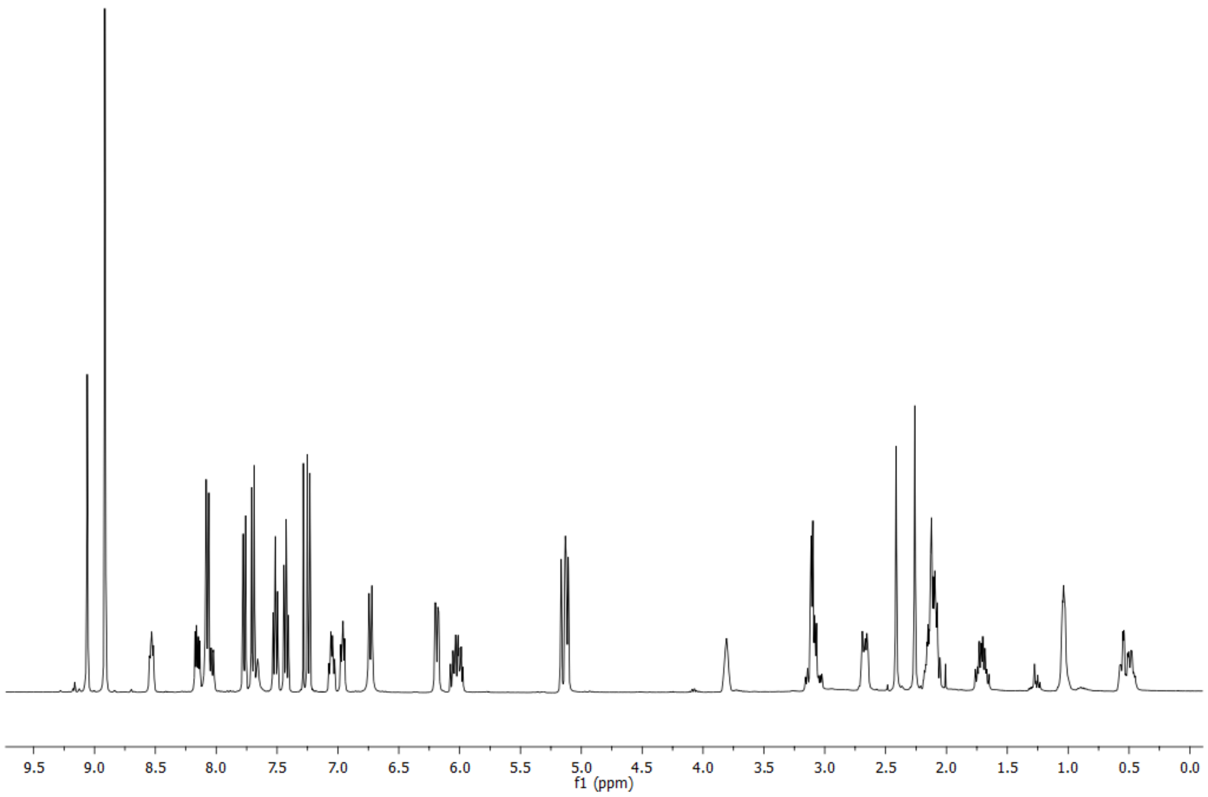


1HNMR Oxcarbazepine

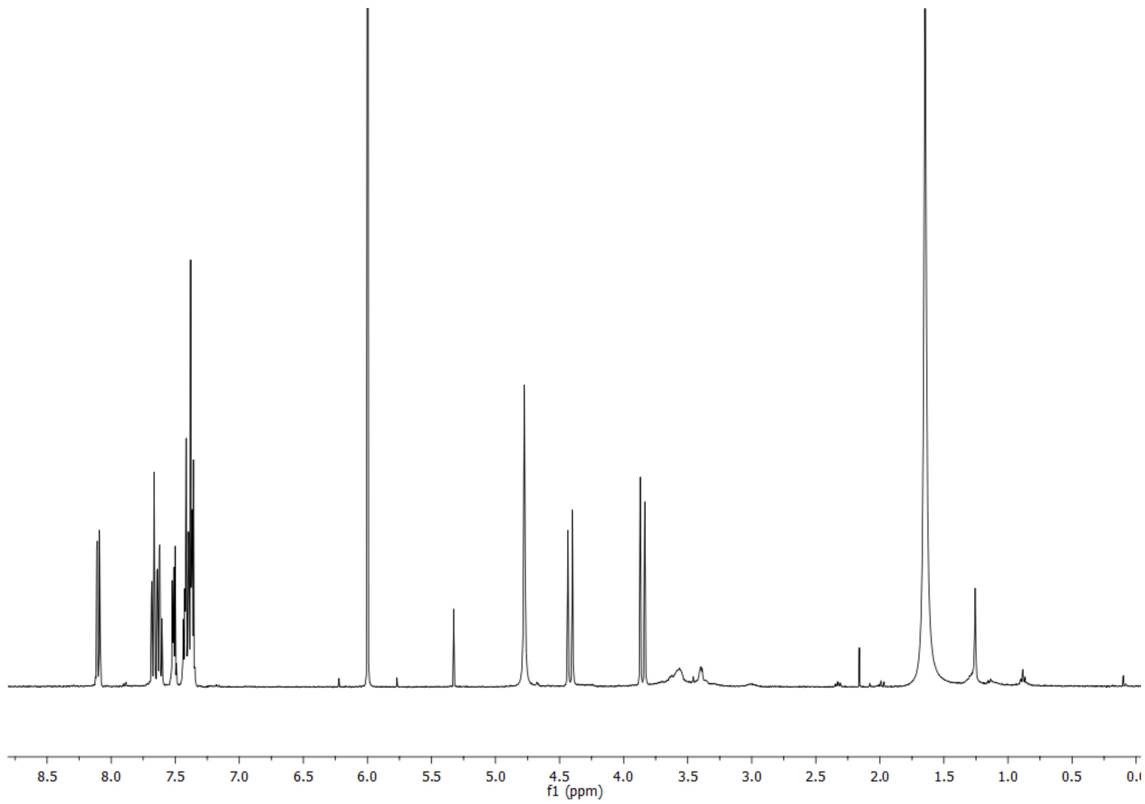


VT-NMR of oxcarbazepine

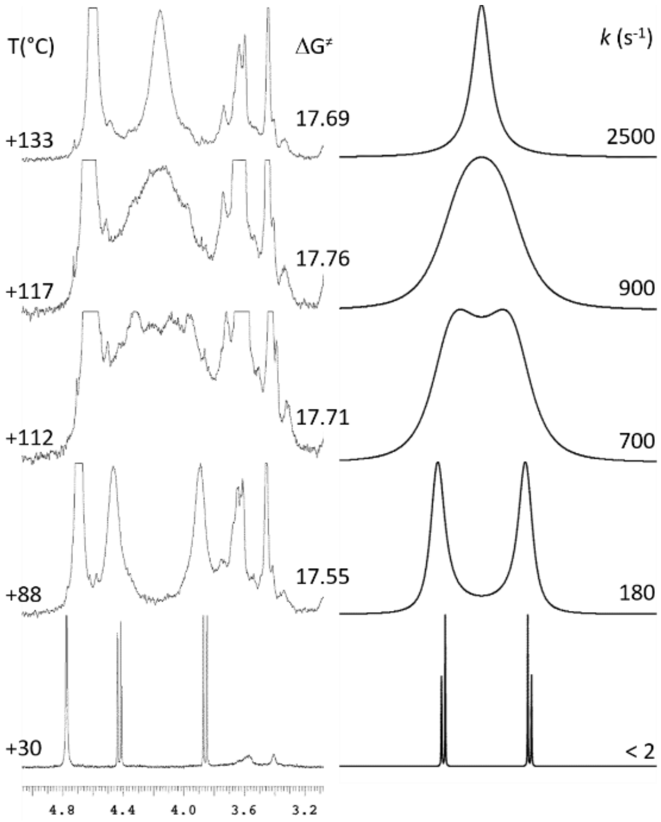

## 2.Simulated VT-HPLC profiles

### 2.1 Nevirapine

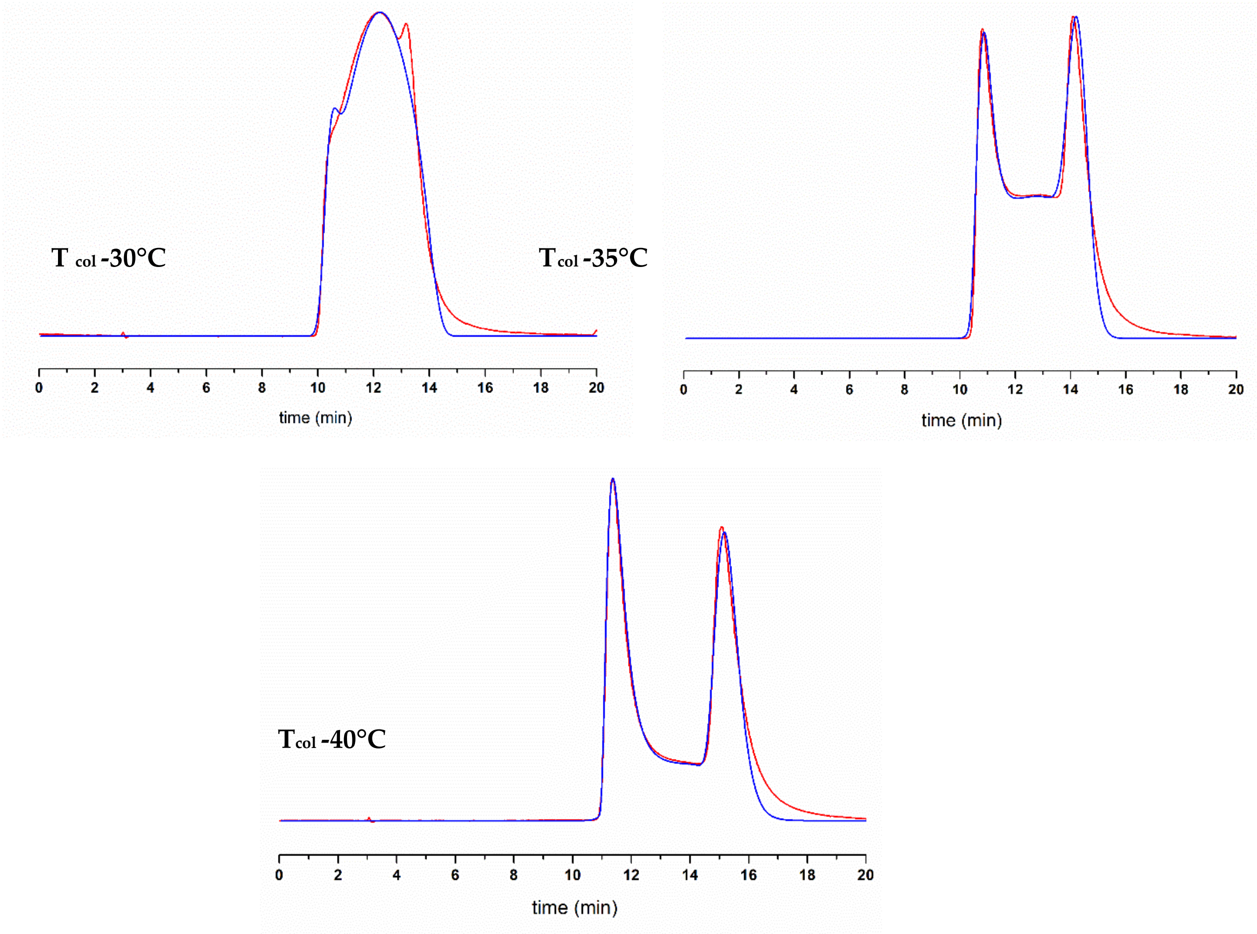


| T°C | $k_{app\ 1,2}$ ($min^{-1}$) | $k_{app\ 2,1}$ ($min^{-1}$) | $\Delta G^{\ddagger}_{app\ 1,2}$ (kcal/mol) | $\Delta G^{\ddagger}_{app\ 2,1}$ (kcal/mol) |
|---|---|---|---|---|
| -30 | 0,231 | 0.171 | 16.82 | 16.96 |
| -35 | 0.073 | 0.055 | 17.01 | 17.14 |
| -40 | 0.076 | 0.057 | 17.08 | 17.22 |

## 2.1 Oxcarbazepine

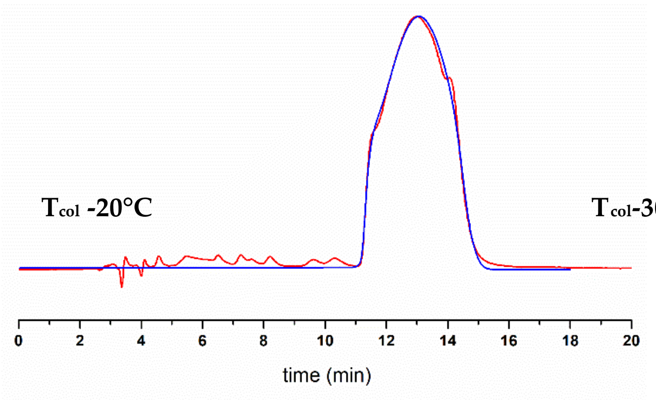


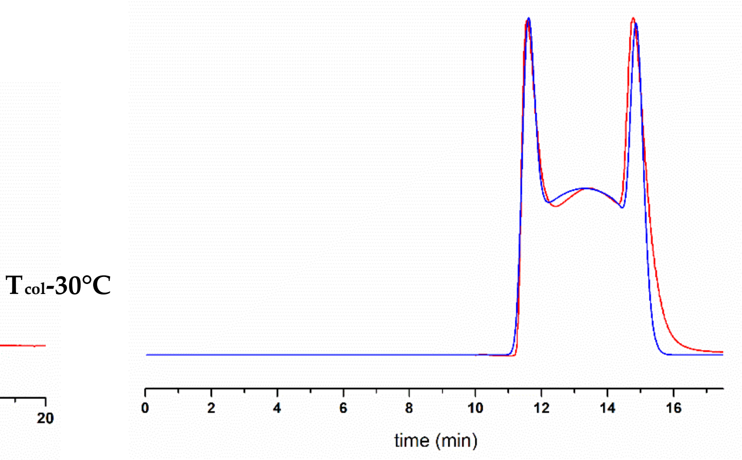


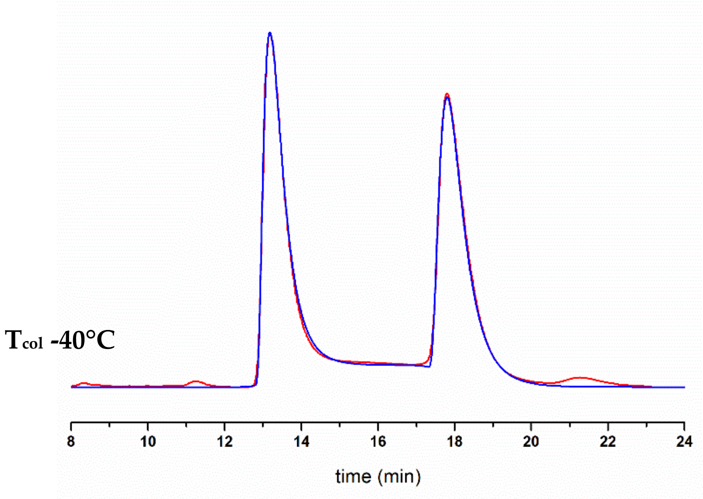


| T°C | $k_{app\ 1,2}$ ($min^{-1}$) | $k_{app\ 2,1}$ ($min^{-1}$) | $\Delta G_{app}^{\ddagger}{}_{1,2}$ (kcal/mol) | $\Delta G_{app}^{\ddagger}{}_{2,1}$ (kcal/mol) |
|---|---|---|---|---|
| -20 | 0.216 | 0.196 | 17.55 | 17.68 |
| -30 | 0.096 | 0.075 | 17.45 | 17.45 |
| -40 | 0.014 | 0.010 | 17.39 | 17.53 |

**3.Geometries of ground state and transition state for NVP and OXC**

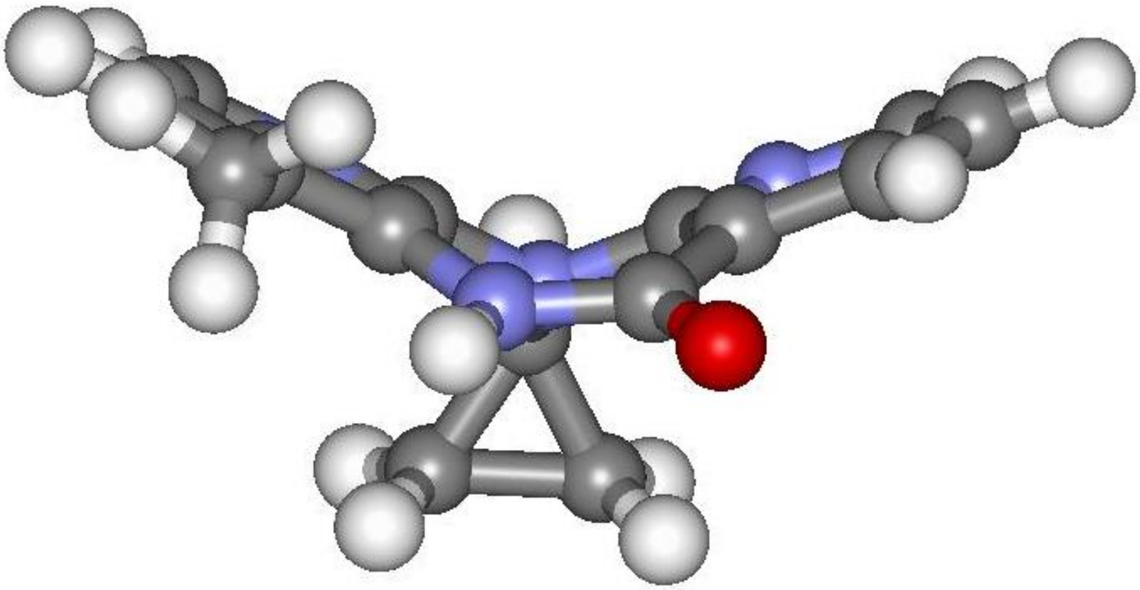

**Ground state geometry found for Nevirapine NVP**

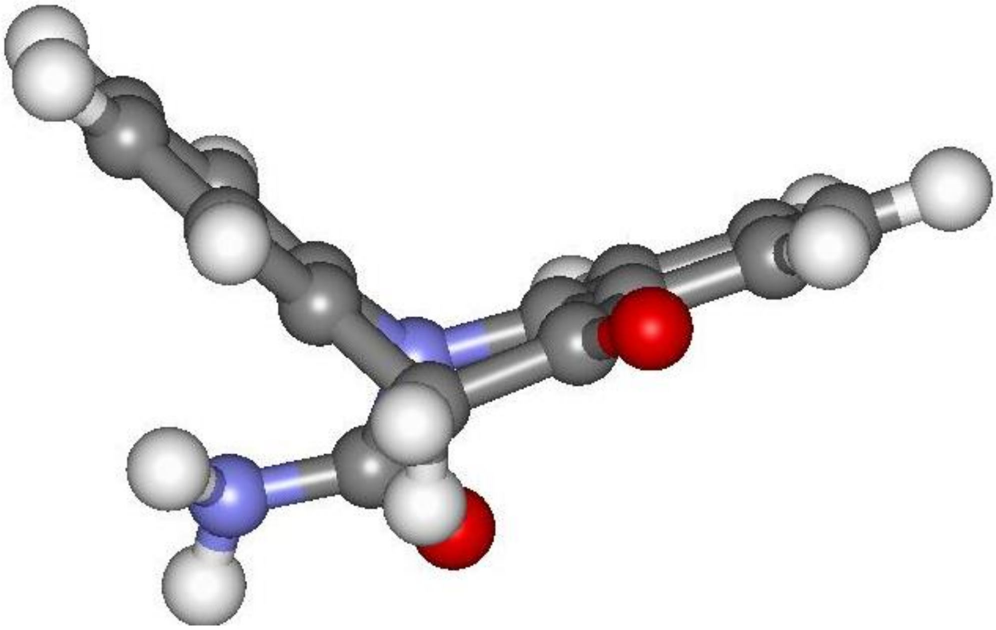

**Ground state geometry found for Oxacarbazepine OXC**

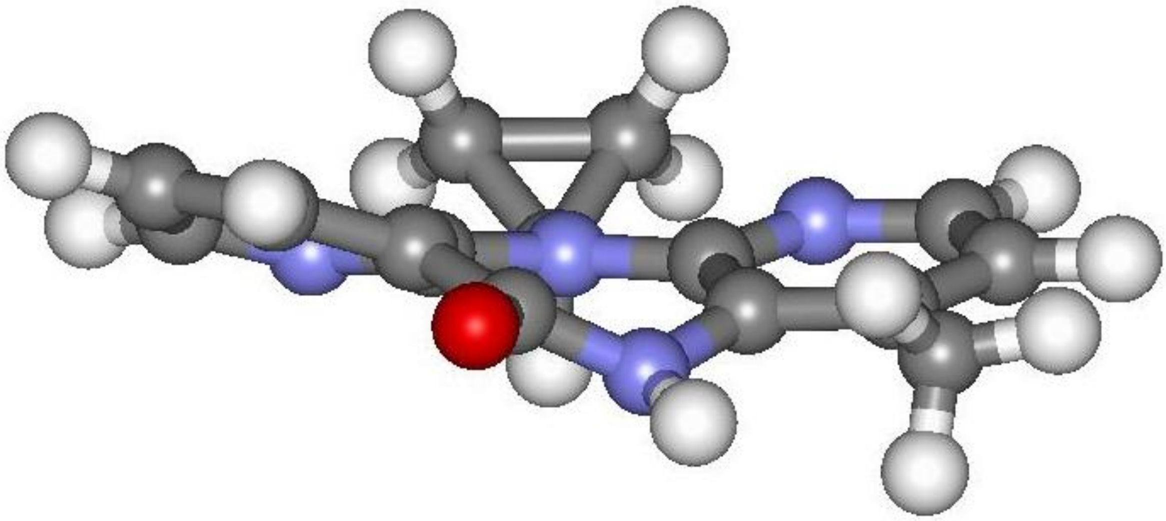

**Transition state geometry found for Nevirapine  NVP enantiomerization**

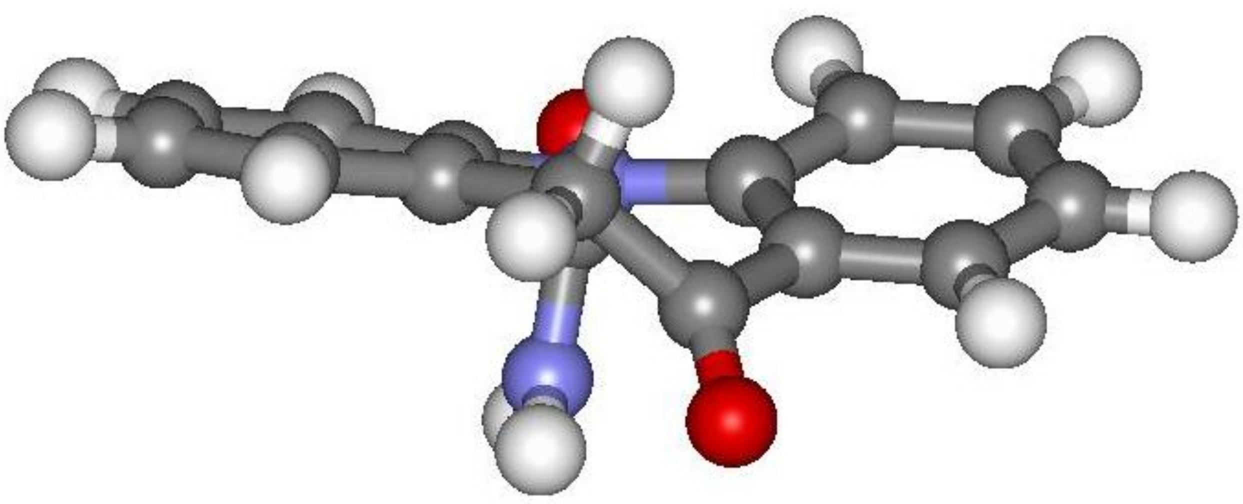

**Transition state geometry found for Oxacarbazepine  OXC enantiomerization**

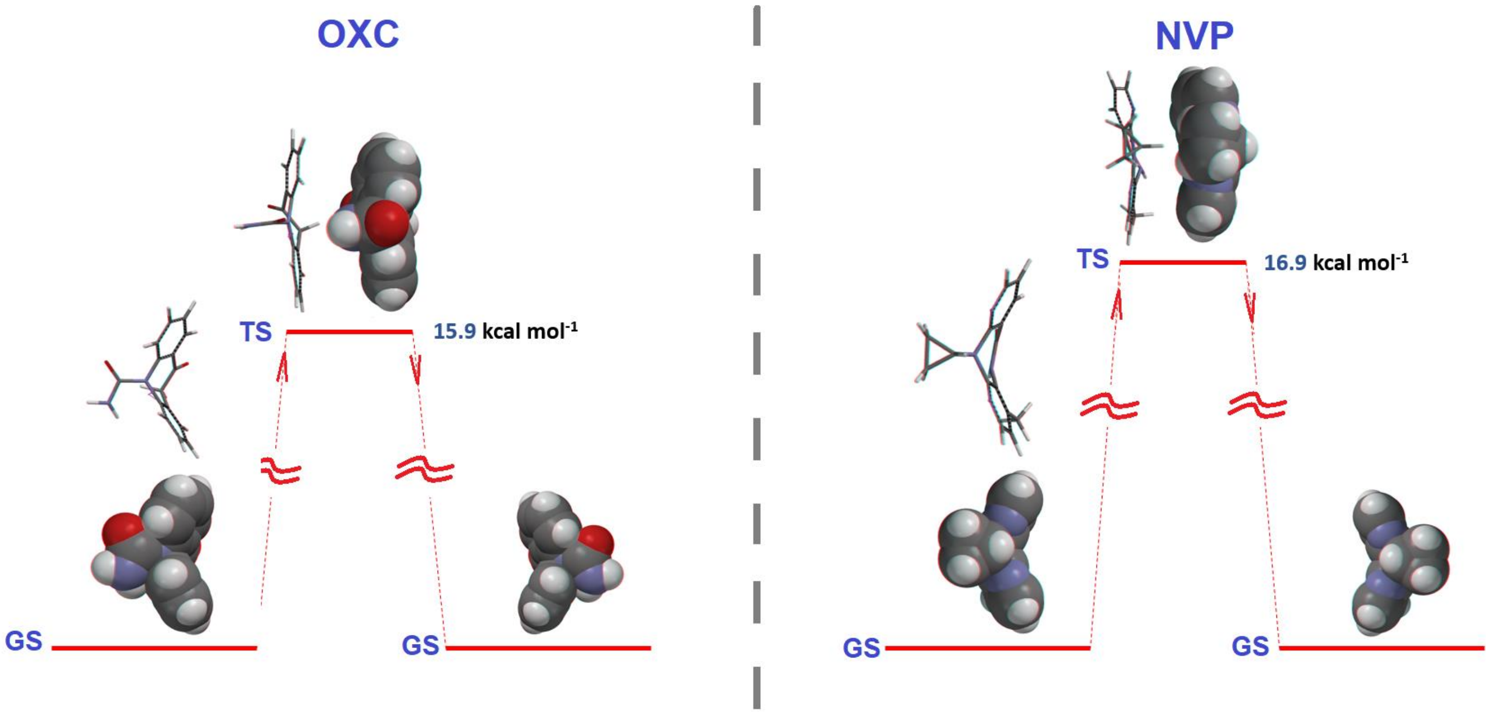


**Enantiomerization barriers found for Oxacarbazepine OXC (left) and Nevirapine NVP (right)**
